# EHMTI-0301. Effect of experimental tooth clenching on the release of beta-endorphin

**DOI:** 10.1186/1129-2377-15-S1-C13

**Published:** 2014-09-18

**Authors:** A Dawson, L Ljunggren, M Ernberg, P Svensson, T List

**Affiliations:** 1 Department of Orofacial Pain and Jaw Function, Faculty of Odontology Malmö University, Malmö, Sweden; 2 Department of Biomedical Laboratory, Faculty of Health Sciences Malmö University, Malmö, Sweden; 3 Section of Orofacial Pain and Jaw Function, Department of Dental Medicine Karolinska Institutet, Huddinge, Sweden; 4 Section of Clinical Oral Physiology, Department of Dentistry Aarhus University and Center for Functionally Integrative Neuroscience (CFIN) MindLab Aarhus University Hospital, Aarhus, Denmark

## Introduction

Several etiologic factors have been suggested for tooth grinding and clenching, but the exact mechanism is not known. One biologic explanation might be that tooth clenching activates the reward system as observed in other types of muscle exercises

## Aims

To investigate the association between experimental tooth clenching and the release of β-endorphin in patients with myofascial temporomandibular disorders (M-TMD) and healthy subjects.

## Methods

Fifteen M-TMD patients and 15 healthy subjects were included and assigned an experimental clenching-task. Venous blood was collected and pain intensity was noted on a visual analog scale. The masseter pressure pain threshold (PPT) was assessed 2-hours before the clenching-task and immediately after. A mixed-model analysis of variance was used for statistical analyses.

## Results

Significant main effects for time and group were observed for pain intensity and PPT, with a significantly higher pain intensity (P < .001) and a significantly lower PPT (P < .01) after the clenching-task compared with baseline. M-TMD patients had significantly higher pain intensity (P < .001) and significantly lower PPT (P < .05) than healthy subjects. No significant time or group effects were observed for the level of β-endorphin. Neither pain intensity nor PPT correlated significantly with β-endorphin levels.

## Conclusions

This experimental clenching-task was not associated with significant alterations in β-endorphin levels over time, but with mechanical hyperalgesia and low to moderate levels of pain in healthy subjects and M-TMD patients, respectively. More research is required to understand the role of the β-endorphinergic system in the etiology of M-TMD.

No conflict of interest.

